# The Wearable VOSTARS System for Augmented Reality-Guided Surgery: Preclinical Phantom Evaluation for High-Precision Maxillofacial Tasks

**DOI:** 10.3390/jcm9113562

**Published:** 2020-11-05

**Authors:** Laura Cercenelli, Marina Carbone, Sara Condino, Fabrizio Cutolo, Emanuela Marcelli, Achille Tarsitano, Claudio Marchetti, Vincenzo Ferrari, Giovanni Badiali

**Affiliations:** 1 eDIMES Lab—Laboratory of Bioengineering, Department of Experimental, Diagnostic and Specialty Medicine, University of Bologna, 40138 Bologna, Italy; emanuela.marcelli@unibo.it; 2 Information Engineering Department, University of Pisa, 56126 Pisa, Italy; marina.carbone@endocas.unipi.it (M.C.); sara.condino@endocas.unipi.it (S.C.); fabrizio.cutolo@endocas.unipi.it (F.C.); vincenzo.ferrari@unipi.it (V.F.); 3 Maxillofacial Surgery Unit, Department of Biomedical and Neuromotor Sciences and S. Orsola-Malpighi Hospital, University of Bologna, 40138 Bologna, Italy; achille.tarsitano2@unibo.it (A.T.); claudio.marchetti@unibo.it (C.M.); giovanni.badiali@unibo.it (G.B.)

**Keywords:** augmented reality, 3D technologies, head-mounted display, virtual planning, 3D printing, maxillofacial surgery

## Abstract

Background: In the context of guided surgery, augmented reality (AR) represents a groundbreaking improvement. The Video and Optical See-Through Augmented Reality Surgical System (VOSTARS) is a new AR wearable head-mounted display (HMD), recently developed as an advanced navigation tool for maxillofacial and plastic surgery and other non-endoscopic surgeries. In this study, we report results of phantom tests with VOSTARS aimed to evaluate its feasibility and accuracy in performing maxillofacial surgical tasks. Methods: An early prototype of VOSTARS was used. Le Fort 1 osteotomy was selected as the experimental task to be performed under VOSTARS guidance. A dedicated set-up was prepared, including the design of a maxillofacial phantom, an ad hoc tracker anchored to the occlusal splint, and cutting templates for accuracy assessment. Both qualitative and quantitative assessments were carried out. Results: VOSTARS, used in combination with the designed maxilla tracker, showed excellent tracking robustness under operating room lighting. Accuracy tests showed that 100% of Le Fort 1 trajectories were traced with an accuracy of ±1.0 mm, and on average, 88% of the trajectory’s length was within ±0.5 mm accuracy. Conclusions: Our preliminary results suggest that the VOSTARS system can be a feasible and accurate solution for guiding maxillofacial surgical tasks, paving the way to its validation in clinical trials and for a wide spectrum of maxillofacial applications.

## 1. Introduction

To date, clinical esthetic evaluation, cephalometric tracings and occlusal splints are the most currently used combination of techniques in performing orthognathic surgery [1,2,3]. These approaches offer the great advantage of being easy, rapid and cost-effective. However, the accuracy is limited by multiple steps requiring a combination of different data sources, including physical examination, anthropometric analysis and bi-dimensional (2D) plain cephalograms, facebow registration and mock surgery using a semiadjustable articulator [4].

In the last two decades, new tools based on three-dimensional (3D) imaging and advanced digital technologies have become available to surgeons during pre-, intra- and postoperative phases of an intervention in the cranio-maxillofacial field [4,5,6,7,8], as well as in other surgical specialties, such as neurology, urology, vascular surgery, orthopedics [9,10,11,12,13,14,15,16,17].

One of the most promising technologies in cranio-maxillofacial surgery is computer-aided design/computer-aided manufacturing (CAD/CAM), which allows the transfer of a patient-specific virtual planned intervention (e.g., bone reconstruction, displacement or resection) to the operating room by using custom-made implants and surgical cutting guides [4,18,19,20].

Another computer-assisted surgery (CAS) modality which is well-established in cranio-maxillofacial, as well as in orthopedics and neurosurgery, is computer-assisted navigation using an external device, the surgical navigator (SN), located outside the operating field [21,22]. The SN is able to detect the patient position and make a precise spatial correlation between the patient and his radiological images or digital reconstructions, indicating to the surgeon the exact position of the anatomical structures, and virtually displaying the position of the surgical instrument. SN-assisted surgery has shown greater accuracy than the occlusal splint [23,24,25,26,27]. However, it also implies significant costs and a complexity of use and management. In addition, an intrinsic limitation is that the surgeon has to perform frequent hand–eye transformations while switching his own attention back and forth between the real surgical field and the navigation data presented on the external display of the SN. This limitation can be overcome with wearable head-mounted displays (HDMs) that give the surgeon an augmented reality (AR) visualization while operating on the patient. AR is a technology that superimposes a computer-generated virtual scenario atop an existing reality, allowing synchronized observation of the digital information and the real surgical field.

A new wearable head mounted display (HMD) based on AR (Video and Optical See-Through Augmented Reality Surgical System, VOSTARS) has been recently developed within the European project VOSTARS (Project ID: 731974, [28]) as an advanced navigation tool for surgery of the facial skeleton and other non-endoscopic surgeries [29,30,31].

Before using VOSTARS on real patients, the system is required to be tested on phantom to evaluate its main functions and usability in a real clinical setting, as well as to quantify the accuracy in performing the same surgical tasks planned for clinical trials.

The purpose of this study is to present the results of in vitro phantom tests performed with the VOSTARS device, notably to evaluate the system feasibility and accuracy in guiding Le Fort 1 osteotomy, which is a typical task in maxillofacial surgery.

Based on the results collected on the phantom model, follow-up clinical applications of the VOSTARS AR guidance will be planned and performed in the near future. These applications may span a wide range of maxillofacial surgical tasks and procedures, including orthognathic surgery, malformations, tumor and reconstructive surgery.

## 2. Materials and Methods

The 10 subjects (7 females and 3 males, aged between 25 and 42) involved in the in vitro tests were the technical employees of EndoCas Centre, University of Pisa, i.e., the main developers and researchers involved in the device design and manufacturing. No written consent was taken from them, but they essentially were fully aware of the device features and of the test goals, and they consented to participate in this phantom test.

For the upcoming clinical use of the device on patients we have received formal ethical approval (VOSTARS-MF.01, 658/2018/Disp/AOUBo) from our Institution (CE-AVEC, S.Orsola-Malpighi Hospital, Bologna, Italy).

### 2.1. VOSTARS Head-Mounted Display

A working prototype of the VOSTARS AR headset was used for the tests. The new wearable AR headset is capable of deploying both video-see-through (VST) and optical see-through (OST)-based augmentations, and it has been specifically designed to be a functional, ergonomic and reliable tool for guiding high-precision manual tasks, as those on facial bones involved in cranio-maxillofacial and plastic surgery [32,33]. The VOSTARS HMD, once worn, is able to provide the surgeon’s eyes with information useful to guide the surgical procedure, such as patient anatomical imaging, location of pathological areas or preoperative plan. The surgeon can select the type of visualization modality (OST/VST) to view the virtual content, according to the specific surgical task to be performed. Under the VST modality, the system can offer an accurate registration between digital and real data, while under the OST modality, the surgeon can take advantage of a natural view.

**Figure 1:**
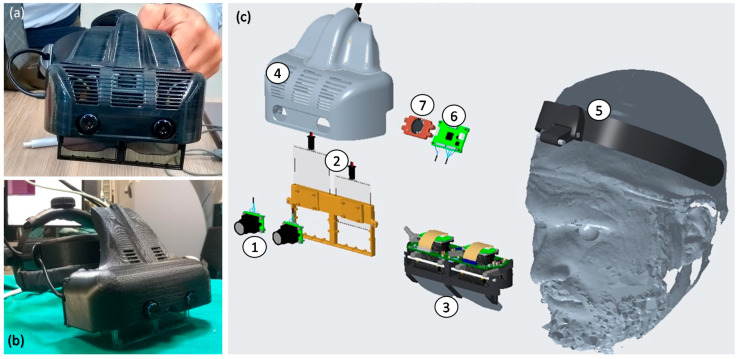
Photos of the assembled Video and Optical See-Through Augmented Reality Surgical System (VOSTARS) headset used for the phantom tests (**a**,**b**), and its exploded view (**c**): (**1**) pair of stereo Red-Green-Blue (RGB) cameras for optical tracking and for video-see-through (VST) augmented reality (AR) visualization; (**2**) pair of optical shutters (OS) for optical see-through (OST)/VST switching; (**3**) see-through microdisplays of the OST Triviso visor; (**4**) plastic cover that holds all the components added around the OST Treviso visor; (**5**) head mount; (**6**) electronic board for stereo synchronization of the camera frames; (**7**) cooling fan.

The system is composed of a hardware framework consisting of a custom-made hybrid OST/VST HDM (Figure 1), and a software framework, both described in detail in our previous works [32,33].

The overall hardware framework (Figure 1) was designed with the aim of mitigating relevant perceptual conflicts typical of commercial AR headsets for close-up activities and ensuring a sufficient stereo overlap at about 40–45 cm, which is an average working distance for surgical tasks.

The HMD is connected to an external computing unit mainly used to control the hardware components and to run the AR software framework. A graphical user interface (GUI) consisting of a panel Personal Computer (PC) is used to set the visualization modality and the rendering of digital contents. A foot-operated pedal, connected to the HMD, is also provided to allow the surgeon to directly command the OST/VST switching, thus passing immediately to a natural view during the surgical procedure.

In detail, the headset is provided with dual SXGA OLED (organic light emitting diode) panels (microdisplays) with 1280 × 1024 resolution, a diagonal field-of-view (FOV) of 30° and an eye relief of 3 cm each. The two panels are controlled by the dedicated computing unit via High-Definition Multimedia Interface (HDMI), and the angular resolution of the OST display is approximately 1.11 arcmin/pixel. The stereo camera pair is composed by two LI-OV4689 cameras by Leopard Imaging, both equipped with a 1/3’’ OmniVision CMOS 4M pixels sensor (pixel size of 2 µm) and with a M12 lens support whose focal length (f = 8 mm) is chosen to ensure a sufficient camera FOV able to cover the entire display FOV at 40 cm. The associated angular resolution of the cameras is approximately 3.6 arcmin/pixel.

The key functions of the software framework are:

(1) A dedicated optical self-tracking mechanism: the head-anchored RGB cameras used for implementing the VST augmentation also provide the stereo localization of three spherical markers (Ø = 11 mm) conveniently placed on the patient’s body and/or around the working area, thus, without requiring obtrusive external trackers or additional tracking cameras [33].
(2) An automatic image-to-patient registration strategy: based on the use of an occlusal splint that embeds the three optical markers. The positions of the optical markers are dictated in the reference system of the Computed Tomography (CT) dataset. By determining their position with respect to the tracking camera (point 1), the pose of the 3D virtual planning can be directly computed in a closed-form fashion by solving a standard absolute orientation problem with three points (i.e., estimating the rigid transformation that aligns the two sets of corresponding triplets of 3D points).
(3) Scene augmentation in both OST and VST modalities: under OST mode, only the computer-generated elements are rendered onto the two microdisplays of the visor, whereas under VST mode, the real views of the world are grabbed by the external RGB cameras and the virtual elements are digitally added to them before the augmented frames are rendered on the two microdisplays.

### 2.2. Experimental Set-Up

#### 2.2.1. Maxillofacial Phantom

**Figure 2:**
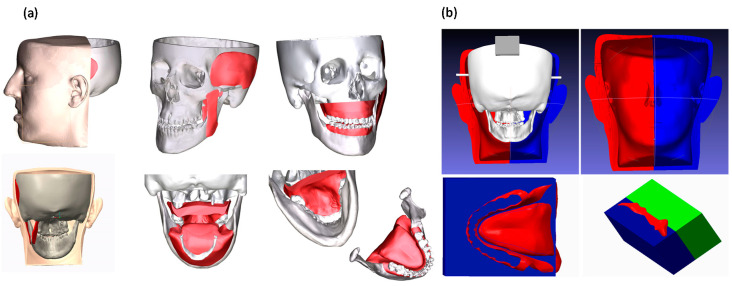
(**a**) 3D virtual model of skull, muscles, soft tissues and facial skin designed for producing the maxillofacial phantom starting from real patient imaging. (**b**) Molds designed for producing the soft components by silicone casting.

**Figure 3:**
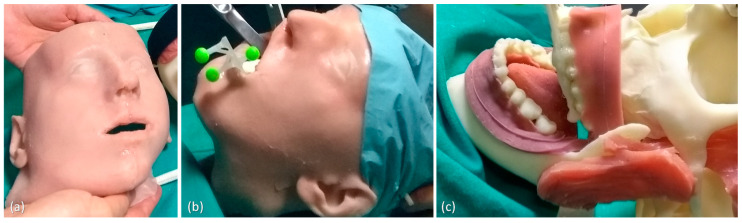
The produced maxillofacial phantom: (**a**) the simulated skin; (**b**) the phantom in use with surgical tools; (**c**) detail of the reproduced teeth and gums.

For the tests, a 3D-printed replica of human facial anatomy (“maxillofacial phantom”) was designed and produced. Starting from real computed tomography datasets, the cranial, maxillary and mandibular bones were extracted with a semiautomatic segmentation pipeline [34] and a complete 3D virtual model of the skull was obtained (Figure 2). From the virtual model, a tangible phantom made of acrylonitrile butadiene styrene (ABS) was produced via 3D printing (3D printer Dimension Elite, Stratasys, Eden Prairie, MN, United States). The primary muscles of mastication (i.e., temporalis, medial pterygoid, lateral pterygoid and masseter) were added to the skull virtual model using Blender software, together with facial soft tissues (soft palate, tongue, gums), functional to the realistic simulation of the surgical procedure (Figure 2). To obtain the physical replicas of the muscles and soft tissues, ad hoc molds were designed, and 3D printed, then silicone casting was made using these molds. Finally, to achieve further realism, and to help keep the jaws in position, facial skin was also designed and produced using the silicone casting technique described above (Figure 2). The resulting maxillofacial phantom is depicted in Figure 3.

#### 2.2.2. Task-Oriented Tracker

**Figure 4:**
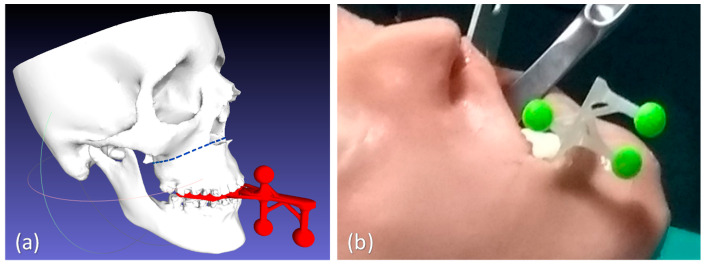
The virtual (**a**) and the 3D-printed model (**b**) of the task-oriented maxillofacial tracker designed for providing optimal tracking and registration during Le Fort 1 osteotomy task.

The accurate AR overlay of the virtual content to the real scene can be achieved by means of a tracking modality that relies on the real-time spatial localization of three reference markers. For the surgical task involving the facial skeleton, the working area is that proximal to the mouth, we designed a CAD template for the tracker with three spherical markers (11 mm in diameter) to be anchored to the occlusal splint typically used as an intraoperative guide in orthognathic procedures (Figure 4). The three spheres were disposed along the sagittal plane in a way to be clearly visible by the front-facing cameras when the surgeon performs the osteotomy task on each face side. The virtual tracker was 3D printed in ABS and the spherical markers were dyed in fluorescent green to boost the response of the RGB camera sensor and improve the robustness of the marker detection under uncontrolled lighting conditions (Figure 4).

### 2.3. Test Execution

#### 2.3.1. VOSTARS Set-Up and Basic Functionality

The experimental set-up was prepared in the operating room: the maxillofacial phantom was positioned on the surgical table, so as to replicate the real position of the patient with respect to the surgeon’s point of view. The VOSTARS headset was adjusted on the surgeon head and the phantom was prepared with the maxilla tracker anchored to the upper dental arch.

The robustness of the optical tracking via the green spherical markers of the maxilla tracker was tested under different levels of illumination of the scialytic lamp (KARL STORZ SE & Co. KG, Tuttlingen, Germany).

Sample virtual contents (e.g., dental profiles, reference target planes) were displayed via the VOSTARS headset both in VST and OST modality. The functioning and usability of the OST/VST switching mechanism controlled by an assistant via the GUI or directly by the surgeon via the foot pedal were experienced.

Finally, a virtual reconstruction of the anatomical facial structures such as bones, nerves, vessels, organs (i.e., parotid gland, tumor mass in the gland) was loaded in the AR software framework and displayed to the surgeon in VST modality. Such 3D reconstructions were obtained by segmentation and 3D modeling of the anatomical structures of interest starting from real patient imaging data (CT scan and Magnetic Resonance Imaging (MRI). The registration (i.e., alignment) of such virtual objects to the real phantom was achieved by means of the said optical tracking relying on the real-time spatial localization of the green spheres anchored to the occlusal splint.

#### 2.3.2. AR-Guided Le Fort 1 Osteotomy Task

The Le Fort 1 osteotomy is a procedure used by surgeons to correct a wide range of dentofacial deformities. Because of its versatility and simplicity, it has gained popularity for a wide range of uses.

Creo Parametric software (7.0, PTC Inc., Boston, MA, USA) was used to design the virtual maxillary osteotomy lines (left and right sides) following the surgical planning previously prepared by the surgeon using Simplant O&O (Dentsply Sirona, York, PA, USA). The two osteotomy lines were represented with dashed curves (0.5 mm thickness) and saved as VRML (Virtual Reality Modeling Language) files to be imported by the AR software framework and displayed in VST by the VOSTARS headset.

**Figure 5:**
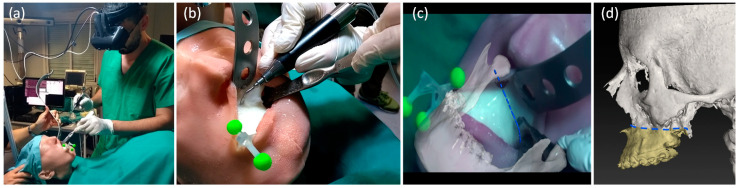
Execution of Le Fort 1 osteotomy under VOSTARS guidance (**a**,**b**), i.e., following the displayed virtual osteotomy line (**c**) derived by preoperative surgical planning (**d**).

The maxilla tracker was mounted on the phantom to track the maxillary segment and to provide registration between virtual content and the real scene. The surgeon, wearing the VOSTARS headset and following the displayed osteotomy lines, performed the Le Fort 1 osteotomy both on the right and left side, using a piezoelectric saw (piezosurgery 3 by Mectron spa, Carasco, Italy; www.piezosurgery.com, accessed on 1 October 2020) typically used in clinical practice (Figure 5a–d, Video S1).

#### 2.3.3. Accuracy Tests

Accuracy tests were carried out to quantify the accuracy level achievable while executing high-precision manual tasks on the skeletal facial anatomy reproduced by the phantom, under the VOSTARS AR guidance. For the tests, we used a similar setting previously adopted by Condino et al. [35] to evaluate the system accuracy in guiding 3D trajectory tracing for generic paths not specifically related to high-precision maxillofacial surgical tasks. In that case, three trajectories (T1–T3) with different degrees of complexity were implemented, i.e., a 2D curve (T1), a 3D curve describing a closed trajectory on a convex surface (T2), a 3D curve describing a closed trajectory consisting of a series of four curves on concave and convex surfaces (T3). For T1–T3, the accurate overlay of the virtual trajectory to the physical 3D-printed model was achieved by real-time tracking of three spherical markers embedded in each model, as previously described [35].

**Figure 6:**
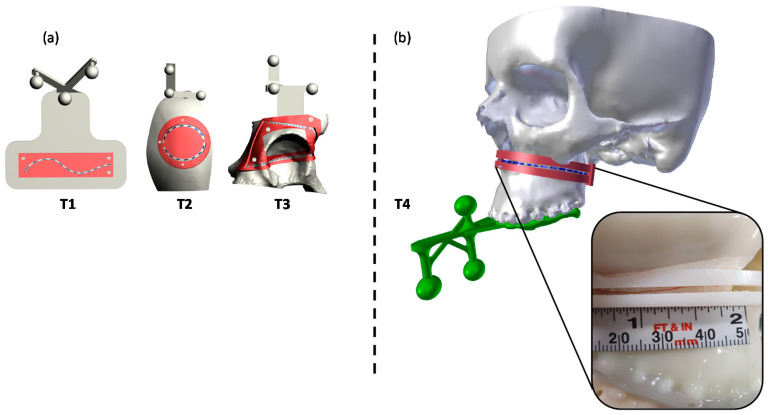
The tested T4 trajectory (**b**), specially related to the Le Fort 1 osteotomy surgical task, and the previously tested generic 3D trajectories T1–T3 (**a**). Trajectories are depicted in blue dashed lines, while the corresponding templates are reported in red, with the inspection window (IW) used to quantify the accuracy level.

For the present study, the considered trajectory (T4) was a 3D curve describing the Le Fort 1 osteotomy line on the maxillofacial phantom. In this case, the tracking for AR overlay was performed via the designed ad hoc maxilla tracker anchored to the occlusal splint (Figure 6).

The same 10 subjects (7 females and 3 males, aged between 25 and 42, technical employees of EndoCas Centre, University of Pisa) who had already performed the accuracy tests on the generic trajectories [35] were involved for the test accuracy of tracing T4 trajectory on the maxillofacial phantom.

Each subject wearing the AR headset was instructed that the primary goal of the test was to accurately trace a line following each trajectory displayed by VOSTARS in VST modality.

A 0.5 mm pencil was used to draw the perceived trajectory on an adhesive tape applied over the model surface. The accuracy of the traced trajectory was evaluated using a template designed ad hoc to match the surface of the phantom model. The template was provided with an inspection window (IW) shaped as the ideal trajectory to be traced. Templates with IW of different widths were designed and 3D printed in ABS, to evaluate two levels of accuracy: ±1.0 mm and ±0.5 mm.

Indeed, the “±1 mm” level of accuracy corresponds to a range of error ± 1 mm, around the line drawn with the 0.5 mm-thick pencil; therefore, a template with an inspection window (IW1) of 2.5 mm (=1 mm + 1 mm + 0.5 mm) was used to evaluate this level of accuracy.

The “±0.5 mm” level of accuracy corresponds to a range of error ± 0.5 mm around the line drawn with the 0.5 mm-thick pencil; in this case, a template with an inspection window (IW2) of 1.5 mm (i.e., 0.5 mm + 0.5 mm + 0.5 mm) was used.

A millimeter adhesive tape was associated to the template and used to measure the cumulative length of the traced line falling within the IW. Then, the percentage of traced trajectories falling within each IW (“percentage accuracy”) was calculated for the tested trajectory (Figure 6).

Moreover, as in the previous work [35], the success ratio (defined as the percentage ratio of the tasks in which the traced trajectory was within a ±2 mm error over the total number of tests performed) was considered.

#### 2.3.4. Statistics

The results of percentage accuracy were reported as mean values and standard deviation (SD). Results obtained for the T4 trajectory were compared with the previously collected measurements for T1–T3 trajectories. For multiple comparison of the mean percentage accuracy obtained in the 4 different trajectories, the Wilcoxon signed rank test was performed. Starting from a *p*-value of 0.05, the Bonferroni correction was applied, therefore setting the significance cut-off value (*p*’) at *p*/*n* = 0.05/6 = 0.0083, where *n* is the number of comparative tests performed. SPSS Statistics Base 19 software (IBM, Armonk, New York, NY, USA) was used to perform the statistical analysis.

## 3. Results

### 3.1. Results of Functionality/Usability Tests

**Figure 7:**
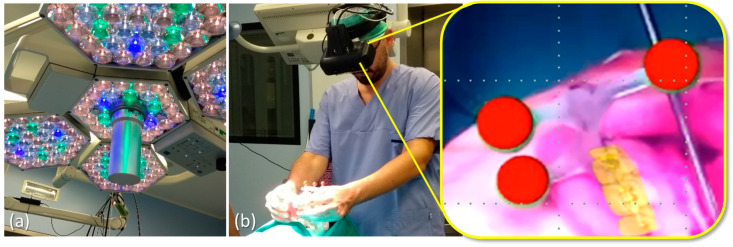
Results obtained under scialitic lamp lighting conditions (**a**) for the tracking and registration using the ad hoc maxilla tracker: red virtual spheres precisely overlaid to the real green ones as seen through the visor (**b**).

VOSTARS set-up in the operating room (OR) was carried out quite easily and quickly. The AR headset showed excellent tracking robustness under OR lighting conditions and a successful registration procedure was also achieved (Figure 7). The surgeon reported good wearability and ergonomics for the headset and judged the foot pedal control for the OST/VST switching to be very useful.

**Figure 8:**
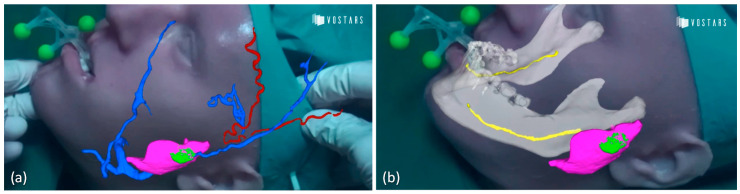
Anatomical augmentation obtained with VOSTARS device displaying vessels with parotid gland and tumor (**a**) and also nerve (**b**). (red: arteries; blue: veins; yellow: nerve; pink: parotid gland; green: tumor mass).

Results of the augmentation of anatomical facial structures experienced by the surgeon with the VOSTARS device are shown in Figure 8 and in Video S2.

### 3.2. Results of AR-Guided Le Fort 1 Osteotomy

The osteotomy task was performed successfully in both facial sides, taking about 2 min per side, therefore, a time comparable to that of a standard execution under natural view. The performed osteotomy was verified using an ad hoc template with an IW of 1.5 mm width, matching the phantom surface: the overall performed osteotomy was within the template IW, thus demonstrating an error lower than ±1 mm in task execution.

### 3.3. Results of Accuracy Tests

The obtained mean values and SD of the percentage accuracy for the T4 trajectory, for both accuracy levels (±1 mm, IW1 = 2.5 mm; ±0.5 mm, IW2 = 1.5 mm) are summarized in Table 1.

As previously reported [35], for generic trajectories (T1–T3), on average the percentage of the trajectory length traced by the subjects with an accuracy level of ± 1.0 mm was 98%, 99% and 94%, respectively. For accuracy level ±0.5 mm, lower percentages resulted (82%, 95% and 83%). Moreover, the success ratio was 100% for T1, 97% for T2, and 80% for T3.

For the Le Fort 1 trajectory (T4), each subject was able to successfully trace the entire trajectory (100% of trajectory length) within an accuracy level of ±1.0 mm and, on average, 88% of the line length within the ±0.5 mm accuracy level. In this case, as for T1, the success ratio was 100%.

For both accuracy levels, the Wilcoxon signed rank test revealed a not statistically significant difference in the accuracy performance between each couple of compared trajectories (Table 2).

The overall less accurate results were for T3, which is probably the more complex 3D trajectory to be traced on a patient acetabular model, which includes many concave and convex surfaces. Regarding T4, good accuracy results were obtained, thus demonstrating that the use of a maxilla tracker anchored to the occlusal splint instead of spherical markers directly attached to the model as in case of T1–T3, does not worsen the accuracy results.

## 4. Discussion and Conclusions

Augmented reality (AR) in surgery has enormous potentialities to help the surgeon in identifying tumor locations, delineating the planned dissection planes, and reducing the risk of injury to invisible structures [36,37,38]. Among possible AR display types, HMDs are emerging as the most efficient and promising medium to support complex manual surgical tasks typically performed under direct vision [36,39], since they allow the surgeon to maintain a “surgeon-centered’ point of view and to leave his/her hands free to operate on the patient.

Nowadays, OST HMDs are the leading wearable AR technology, and several consumer level heads-up displays are entering the market following the success of the HoloLens (Microsoft, Redmond, WA, USA) [40].

Nevertheless, technological and human factor limitations, such as the presence of a small augmentable field-of-view, a low contrast image, and the lack of reliable AR image registration, still hinder their routine use for high-precision manual tasks.

The presented VOSTARS AR HMD is a specially designed solution for accurate close-up works, such as surgical tasks [32,33]. Our first finding was that VOSTARS HMD can be efficiently and readably adopted in a real operating room environment, showing a simple set-up, as well as a robust tracking capability via the ad hoc designed maxilla tracker. The VOSTARS headset fulfilled all the major requirements for a surgery-specific HMD that are related to ergonomics, safety and reliability [36,41].

About ergonomics, the surgeon did not report any specific discomfort after wearing the visor for about 45 min. Some future improvements for the whole system may be considered, such as the possible integration of the computing unit in a backpack or a belt pocket, which would allow the surgeon to move more freely around the operating table.

About safety requirements, the quick and user-controlled mechanism for VST/OST switching ensures the preservation of the natural vision of the surgical field in case of possible fault of the system or whenever the surgeon needs to suddenly remove the AR overlay.

About reliability, our preliminary results on phantoms suggest that the VOSTARS headset can allow accurate guidance in performing the selected surgical tasks. In our previous study [35] in 7/90 trials, the users failed in tracing the line, having an error greater than ±2 mm. In this work, in all 10 trials for T4, the users were able to trace the line within ±2 mm error (100% of success rate), indeed even within ±1 mm error.

The VOSTARS system showed submillimetric accuracy in tracing Le Fort 1 osteotomy trajectories. This represents a successful and encouraging preliminary result, considering 1–2 mm accuracy is regarded as an acceptable range in the context of image-guided surgery [41].

**Figure 9:**
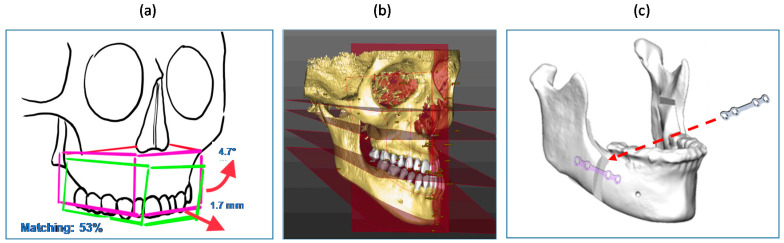
Examples of future virtual content to be displayed by VOSTARS: (**a**) numeric and arrow indicators for guiding bone repositioning; (**b**) cephalometric data and planes; (**c**) osteosynthesis devices.

Future directions should be addressed to transfer the VOSTARS testing in the real clinical field, to assist similar and more complex surgical tasks, e.g., mandibular osteotomies that involve bone cutting along different planes and depths, or maxillary repositioning. Virtual content, like directional arrows or numerical indicators, could be implemented to guide the repositioning movements (Figure 9a). Cephalometric data and anatomical 3D templates of facial hard and soft tissues derived from processing CT scans [10] could also be added as virtual content to give the surgeons additional anatomical references and frames to follow during surgery (Figure 9b). Such virtual textual and reference information could be displayed in the OST modality, since in this case there is no need for an extremely accurate (<1 mm) registration, favoring the advantage of a natural view of the surgical field. Finally, the geometry, shape and position of osteosynthesis devices can be displayed using VOSTARS (Figure 9c) to make them clearly visible to the surgeon relative to the underlying facial structures, thus paving the way for a future “less invasive” open surgery.

The VOSTARS device can have prospective use also in tumor and head and neck reconstructive surgery, e.g., in providing anatomical visualization and in supporting mandibular complex reconstruction. Some previous studies reported experiences of AR-assisted navigation used in combination with CAD/CAM technology to guide the positioning of the fibular osteotomy cutting guide [7], as well as to facilitate anatomical visualization during free flap harvesting [42], but in both cases, the AR guidance was performed using hand-held mobile devices (smartphone or tablet). VOSTARS can represent an improved AR-assisted navigation system that allows the surgeon to view directly in front of his eyes the virtual content superimposed on the patient.

The presented preclinical phantom evaluation, although required to be extended to further and more complex surgical tasks, represents a solid starting point to plan a systematic clinical trial evaluation of the new VOSTARS AR platform for maxillofacial surgical applications, which will happen soon.

## Figures and Tables

**Table 1:** Mean percentage and standard deviation (SD) of the traced lines falling within the inspection window (IW) of the two templates used to evaluate the two accuracy levels (±1 mm, IW1 = 2.5 mm; ±0.5 mm, IW2 = 1.5 mm) for the tested T4 trajectory.

Subject	T4	
	IW1 (±1 mm)	IW2 (±0.5 mm)
1	100.0%	81.4%
2	100.0%	93.8%
3	100.0%	100.0%
4	100.0%	100.0%
5	100.0%	87.6%
6	100.0%	49.5%
7	100.0%	93.8%
8	100.0%	86.6%
9	100.0%	92.8%
10	100.0%	90.7%
Mean	100.0%	87.6%
SD	0.0%	14.6%

IW = inspection window; SD = standard deviation.

**Table 2:** *p’*-Values of the Wilcoxon test performed for multiple comparison.

Accuracy Level.	T1 vs. T2	T1 vs. T3	T1 vs. T4	T2 vs. T3	T2 vs. T4	T3 vs. T4
±1.0 mm	0.465	0.326	0.109	0.115	0.042	0.042
±0.5 mm	0.214	0.386	0.507	0.069	0.508	0.047

*p*’ = *p/n* = 0.05/6 = 0.0083; *n* = number of tests.

## Supplementary Materials

The following are available online at https://www.mdpi.com/2077-0383/9/11/3562/s1. Video S1: Execution of Le Fort 1 osteotomy under VOSTARS guidance. Video S2: Augmentation of anatomical facial structures using the VOSTARS device.
